# Controlling of Bacterial Virulence: Evaluation of Anti-Virulence Activities of Prazosin against *Salmonella enterica*

**DOI:** 10.3390/antibiotics11111585

**Published:** 2022-11-09

**Authors:** Mahmoud A. Elfaky, Abrar K. Thabit, Khalid Eljaaly, Ayat Zawawi, Ahmed S. Abdelkhalek, Ahmad J. Almalki, Tarek S. Ibrahim, Wael A. H. Hegazy

**Affiliations:** 1 Department of Natural Products, Faculty of Pharmacy, King Abdulaziz University, Jeddah 21589, Saudi Arabia; 2 Centre for Artificial Intelligence in Precision Medicines, King Abdulaziz University, Jeddah 21589, Saudi Arabia; 3 Pharmacy Practice Department, Faculty of Pharmacy, King Abdulaziz University, Jeddah 21589, Saudi Arabia; 4 Department of Medical Laboratory Sciences, Faculty of Applied Medical Sciences, King Abdulaziz University, Jeddah 21589, Saudi Arabia; 5 Vaccines and Immunotherapy Unit, King Fahd Medical Research Center, King Abdulaziz University, Jeddah 21589, Saudi Arabia; 6 Medicinal Chemistry Department, Faculty of Pharmacy, Zagazig University, Zagazig 44519, Egypt; 7 Department of Pharmaceutical Chemistry, Faculty of Pharmacy, King Abdulaziz University, Jeddah 21589, Saudi Arabia; 8 Department of Microbiology and Immunology, Faculty of Pharmacy, Zagazig University, Zagazig 44519, Egypt; 9 Pharmacy Program, Department of Pharmaceutical Sciences, Oman College of Health Sciences, Muscat 113, Oman

**Keywords:** antimicrobial resistance, *Salmonella enterica*, virulence, anti-biofilm, prazosin

## Abstract

*Salmonella enterica* is a Gram-negative orofecal transmitted pathogen that causes a wide diversity of local and systemic illnesses. *Salmonella enterica* utilizes several interplayed systems to regulate its invasion and pathogenesis: namely, quorum sensing (QS) and type three secretion system (T3SS). In addition, *S. enterica* could sense the adrenergic hormones in the surroundings that enhance its virulence. The current study aimed to evaluate the ability of α-adrenoreceptor antagonist prazosin to mitigate the virulence of *S. enterica* serovar Typhimurium. The prazosin effect on biofilm formation and the expression of *sdiA*, *qseC*, *qseE*, and T3SS-type II encoding genes was evaluated. Furthermore, the prazosin intracellular replication inside macrophage and anti-virulence activity was evaluated in vivo against *S*. *typhimurium*. The current finding showed a marked prazosin ability to compete on SdiA and QseC and downregulate their encoding genes. Prazosin significantly downregulated the virulence factors encoding genes and diminished the biofilm formation, intracellular replication inside macrophages, and in vivo protected mice. To sum up, prazosin showed significant inhibitory activities against QS, T3SS, and bacterial espionage, which documents its considered anti-virulence activities.

## 1. Introduction

*Salmonella enterica* is a facultative intracellular, invasive, gastrointestinal pathogen, and it causes diverse human diseases ranging from local gastroenteritis to systemic typhoid fever [1,2]. *Salmonella* species are classified into typhoidal and nontyphoidal, and both cause invasive infections and critical enteric fever if not treated properly, leading to higher mortality rates [3,4]. Quorum sensing (QS) is the cell-to-cell communication system in which bacteria employ autoinducers (AIs), such as N-acylhomoserine lactones (AHLs) to play a key role in bacterial virulence [5,6]. *S. enterica* comprises at least two QS types, one is induced by AHLs and the other is induced by autoinducer-2 (AI-2) [6,7,8,9]. QS regulates several bacterial physiological activities including bacterial motility, the expression of virulence factors, and biofilm formation that enhances bacterial virulence and pathogenesis [10,11,12]. However, *S. enterica* does not produce an AHL synthase; instead, it encodes a Lux-type QS receptor homolog SdiA which senses AHLs’ different acyl chain lengths [5,6]. Interestingly, SdiA senses exclusively AHLs synthesized by other bacterial species playing significant roles in controlling valence [6,8].

*S. enterica* employs two different types of type III secretion systems (T3SS), which mediate separate functions [2]. Several important *S. enterica* virulence factors are located in definite loci called Salmonella Pathogenicity Islands (SPI) [1,13,14]. Two main SPI encode T3SS to translocate their effectors in different phases of pathogenesis [15,16,17]. In the early phase, SPI1-T3SS translocates T3SS-type-1 effectors to modify the cellular G-proteins facilitating the invasion. Later, SPI2-T3SS mediates the translocation of effectors needed for intracellular survival [17]. In addition to QS and T3SS, *S. enterica* senses neuroendocrine hormones and other catechol amines in a phenomenon called bacterial espionage [18,19,20,21,22]. Bacterial espionage was observed particularly in gut pathogens, where bacterial sensors sense the surrounding chemical changes, easing their accommodation and enhancing bacterial virulence [23,24]. As a cross-talk between bacteria and host cells, the AIs detected by QS receptors cross-talk with the adrenergic hormones triggering the same signaling pathway to enhance bacterial virulence [20,21,24,25]. Furthermore, bacterial AIs induce neuroendocrine hormone production in host cells, which results in increased bacterial sensation to neuroendocrine hormones exaggerating bacterial virulence [20,26]. There is cumulative evidence that *S. enterica* senses the host neuroendocrine stress hormones, enhancing bacterial virulence [20,21,22,24].

The increasing resistance development is one of the main health issues, especially in serious infections such as *S. enterica* enteric fevers [4,27,28]. The elevated resistance of *S. enterica* to several antibiotics that were assumed effective is observed around the world [28,29]. The dwindled supply of new efficient antibiotics complicates the situation and dictates the innovation of new approaches. As an interesting approach, alleviating bacterial virulence is assumed to help the immune system in the complete eradication of invading bacteria without affecting their growth, avoiding resistance development [30,31,32,33]. For this purpose, several approved drugs were repurposed to serve as efficient anti-virulence agents [34,35,36,37,38,39,40]. Despite the obvious merits of drug repurposing, there are critical concerns that must be resolved to avoid adverse effects before their use as anti-virulence agents [10,41,42,43].

**Figure 1:**
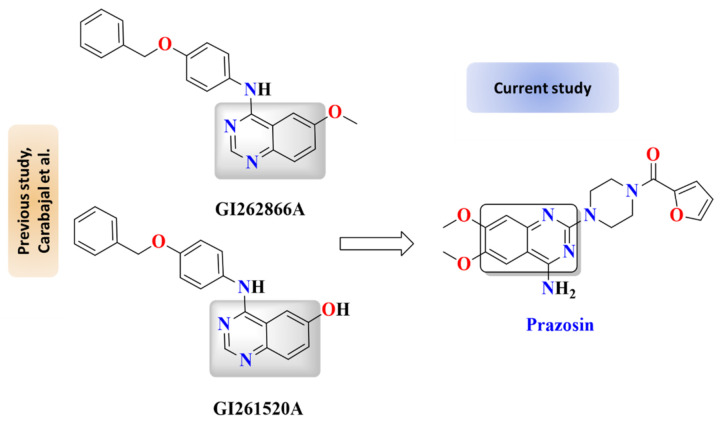
*Salmonella* employs different systems to regulate its virulence. The *Salmonella* PhoP/PhoQ regulatory system and quorum sensing system (QS) regulate the expression of virulence encoding genes, and their targeting could dramatically diminish the bacterial virulence. In addition to bacterial systems, *Salmonella* pathogenesis is enhanced by its ability to spy on the host systems, sensing adrenergic hormones using membranal receptors. It was shown that compounds GI262866A and GI261520A exhibit anti-virulence activities via targeting the *Salmonella* PhoP/PhoQ regulatory system [45]. In the current study, prazosin shares quinazoline moiety with compounds GI262866A and GI261520A, and it can significantly diminish bacterial virulence via targeting QS and preventing bacterial espionage.

*Salmonella* has a two-component regulatory system PhoP/PhoQ that regulates the expression of genes involved in virulence and resistance to host defense antimicrobial peptides. Furthermore, the PhoP/PhoQ system promotes the *Salmonella* internalization inside host immune cells [44]. It was shown that targeting the sensor kinase PhoQ results in the inhibition of *S. typhimurium*. Moreover, *Salmonella* can sense the adrenergic hormones and other catecholamines via membranal sensor kinases as QseC, which results in the augmentation of its virulence [20,22]. Carabajal et al. documented the anti-virulence activity of quinazoline-containing compounds via targeting *S*. *typhimurium* PhoQ sensor kinase [45]. In this context, several approaches were proposed to synthesize pharmacophores that can target bacterial histidine kinases to be employed as anti-virulence agents [45,46,47,48]. Prazosin is an α-adrenoreceptor antagonist that is used widely in the treatment of hypertension and related diseases [49]. Interestingly, prazosin possesses the quinazoline moiety that may indicate its anti-virulence activity (Figure 1).

Bearing in mind the virulence behavior of *S. enterica*, hindering the adrenergic receptor could reduce the bacterial spy on host cells that results in the mitigation of bacterial virulence [20,21]. It was hypothesized that adrenoreceptor blockers are suitable candidates to reduce bacterial espionage [31,32]. Furthermore, α-adrenoreceptor blocker terazosin showed significant anti-QS activities against *Pseudomonas aeruginosa* and *S. enterica* serovar Typhimurium [19]. That is inspiring to further evaluate the anti-virulence activities of other α-adrenoreceptor blockers. It is aimed to in vitro and in vivo evaluate the anti-virulence activities of prazosin against *S. enterica* serovar Typhimurium and *Escherichia coli* K-12, exploring its effects on QS, T3SS, and bacterial espionage.

## 2. Results

### 2.1. Determination of Minimum Inhibitory Concentration (MIC) of Prazosin on S. typhimurium and E. coli K-12

**Figure 2:**
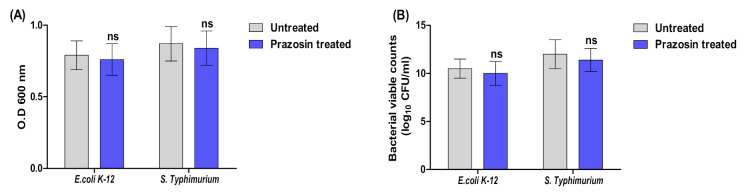
Prazosin at sub−MIC has no effect on bacterial growth. The bacterial cultures were grown in the presence or absence of prazosin. (**A**) The optical densities, (**B**) Bacterial counts were determined, and there were no significant differences between the growth in the presence or absence of prazosin. ns: non-significant.

A broth microdilution method was employed to determine the MICs of prazosin against the tested bacterial stains. The lowest concentrations of the prazosin that inhibited the growth of *S. Typhimurium* and *E. coli* K-12 were 4 and 2 mg/mL, respectively. To avoid any prazosin effect on bacterial growth, all the subsequent tests were performed using prazosin at sub−MIC (1/4 MIC). Furthermore, the bacterial strains were overnight cultured in the presence of prazosin at sub−MIC, and the optical densities and bacterial counts were compared to untreated cultures. Significant differences between treated and untreated cultures with prazosin at sub−MIC were not observed (Figure 2).

### 2.2. Anti-QS and Anti-Biofilm Activities of Prazosin

#### 2.2.1. Prazosin Downregulated the Expression of sdiA Gene

**Figure 3:**
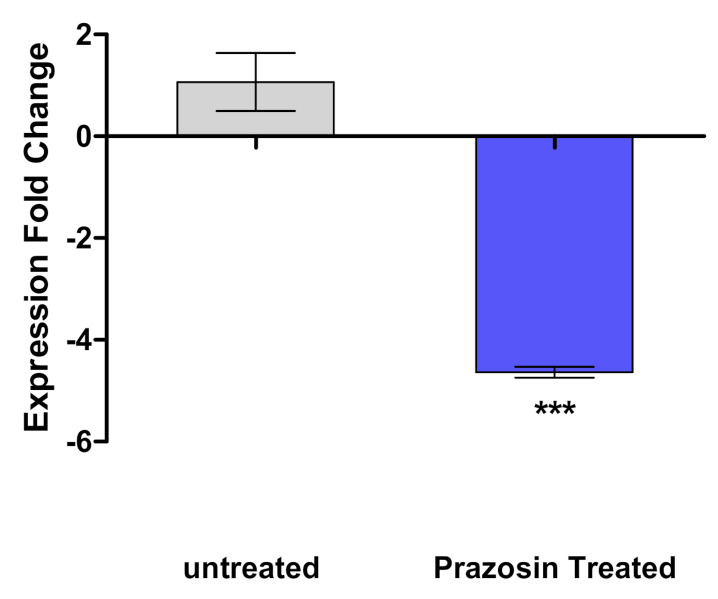
Prazosin downregulated the expression of LuxR homolog QS receptor SdiA. Prazosin significantly reduced the expression of *Salmonella sdiA* gene (*** = *p* < 0.0001).

The expression of *sdiA* gene in *Salmonella* was quantified in the presence and absence of prazosin at sub−MIC. Prazosin significantly reduced the expression of the *sdiA* gene (Figure 3).

#### 2.2.2. Prazosin’s Anti-Biofilm Activity

**Figure 4:**
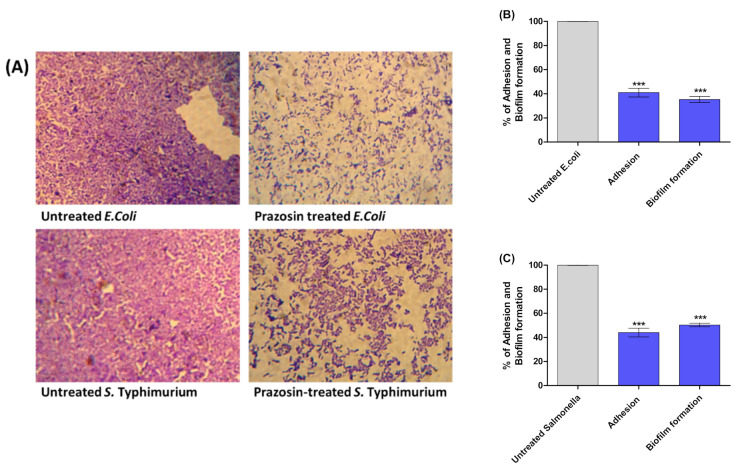
Prazosin diminished bacterial adhesion and biofilm formation. (**A**) Light microscope images represent the anti-biofilm activity of prazosin at sub−MIC. The crystal violet method was employed to evaluate bacterial adhesion and biofilm formation. The percent change in adhered and biofilm-forming (**B**) *E. coli* and (**C**) *S. Typhimurium* was significantly reduced in the presence of prazosin at sub−MIC (*** = *p* < 0.0001).

The QS system plays a key role in the regulation of the bacterial virulence and controls the production of several virulence factors as biofilm formation. In this context, the effects of prazosin on the *S*. *typhimurium* and *E. coli* K-12 adhesion biofilm formation were evaluated. Interestingly, prazosin at sub−MIC diminished bacterial adhesion and biofilm formation on abiotic surfaces (Figure 4).

### 2.3. Prazosin Likely Silences the Bacterial Espionage

Prazosin Downregulated the Expression of qseC and qseE

**Figure 5:**
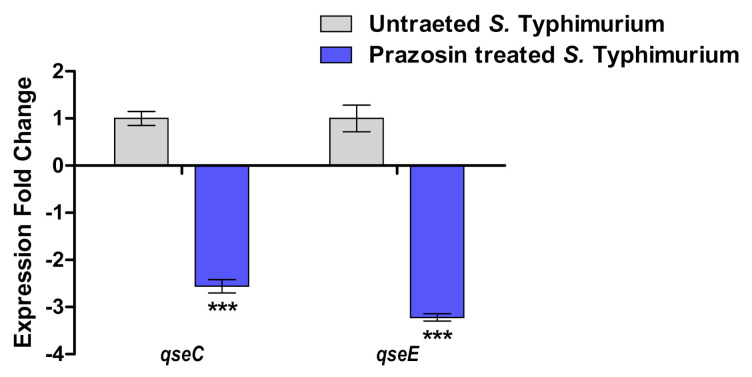
Prazosin interferes with *Salmonella* adrenoreceptor sensors. Prazosin at sub−MIC significantly reduced the expression of membranal adrenoreceptor sensors *qseC* and *qseE* (*** = *p* <0.0001).

For attesting the possible inhibitory effect of prazosin at sub−MIC on bacterial espionage, the expression levels of norepinephrine membranal sensors encoding genes *qseC* and *qesE* were evaluated in the presence of prazosin. Prazosin significantly decreased the expression of both *qseC* and *qseE* in *S*. *Typhimurium* (Figure 5).

### 2.4. Prazosin Interfered with the Intracellular Replication of S. typhimurium

#### 2.4.1. Prazosin Interfered with the *S. typhimurium* Intracellular Replication in Macrophages

**Figure 6:**
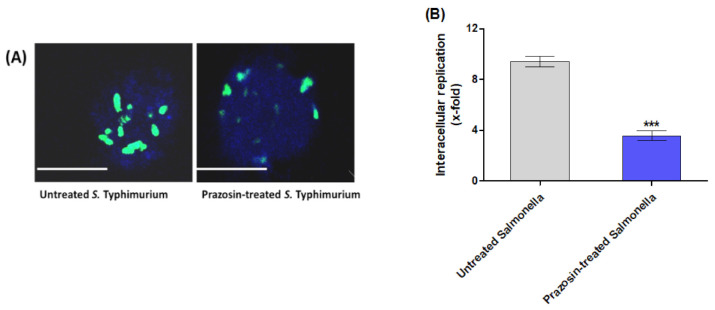
Prazosin decreased *S. Typhimurium* intracellular replication. (**A**) Microscopic images of *S. Typhimurium* in macrophages. Prazosin markedly decreased the intracellular replication of *Salmonella* inside macrophages. (**B**) The phagocytosed cell/relative untaken cell percentage and x-fold intracellular replication were calculated. Prazosin at sub−MIC significantly decreased the intracellular replication of *Salmonella* (*** = *p* < 0.0001). Scale bars correspond to 100 μm.

*S*. *typhimurium* is described as intracellular bacteria that can survive and replicate inside the macrophage phagosome [1,2]. The gentamicin protection assay was employed to evaluate the *Salmonella* internalization within macrophages. The counts of intracellular bacterial replication were calculated as x-fold (16 h against 2 h). Prazosin at sub−MIC significantly decreased the number of surviving bacteria (Figure 6B). For visualization, *Salmonella* cells were immune stained inside macrophage cells (Figure 6A). Clearly, prazosin decreased the intracellular *Salmonella*.

#### 2.4.2. Prazosin Downregulated the Expression of T3SS-Type 2 Encoding Genes

**Figure 7:**
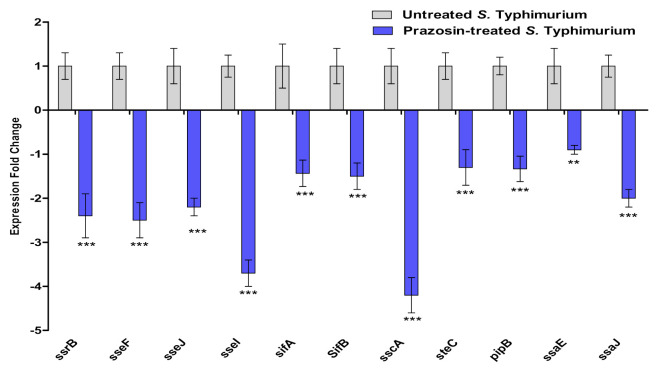
Prazosin decreased the T3SS-type 2 encoding gene expression. Prazosin at sub−MIC significantly decreased the expression of T3SS-type 2 encoding genes (*** = *p* < 0.0001; ** = *p* < 0.001).

*S*. Typhimurium T3SS-type 2 plays a crucial role in its survival inside the phagosome of antigen-presenting cells [15,50]. Interfering with the T3SS-type 2 could lead to the possible inhibition of *Salmonella* intracellular replication. The expression of different T3SS-Type 2 encoding genes in the presence of prazosin at sub−MIC was evaluated in comparison to untreated control. Prazosin significantly diminished the expression of the tested genes (Figure 7).

### 2.5. Prazosin In Vivo Anti-Virulence Activity

**Figure 8:**
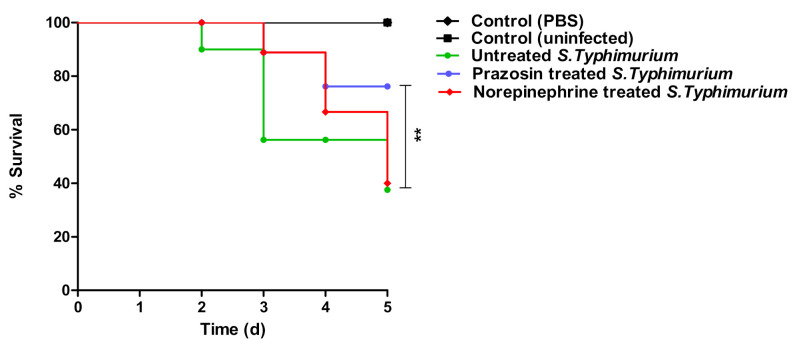
Prazosin in vivo anti-virulence activity. Five mice groups were injected with untreated S. Typhimurium, norepinephrine-treated *S*. *typhimurium*, Prazosin-treated *S*. *typhimurium*, PBS, or kept non-injected. Prazosin significantly decreased the capacity of *S*. *typhimurium* to kill mice (from 50% to 20%), Log-rank test for trend *p* = 0.0048 (** = *p* < 0.001).

In order to evaluate the anti-virulence activity of prazosin, the ability of prazosin at sub−MIC to protect mice against *S*. *typhimurium* was assessed by employing in vivo protection assay. To further support the hypothesis, the effect of norepinephrine on *S*. *typhimurium* was also evaluated. There were no deaths observed in the mice groups injected with sterile PBS or kept uninfected. In the mice groups that were injected with untreated *Salmonella* or norepinephrine-treated *Salmonella*, the survival rate was 50% (5 deaths out of 10 mice). Meanwhile, in the mice injected with prazosin-treated *Salmonella*, the survival rate increased to 80% (2 deaths out 10 mice). These findings support that prazosin significantly diminished the *S. typhimurium* capacity to kill mice (*p* = 0.0048) (Figure 8).

## 3. Discussion

Despite the success of antibiotics at lessening the mortality and morbidity of bacterial infections, bacteria have acquired the ability to become resistant to antibiotics, diminishing their impact [51,52,53,54]. This situation of bacterial resistance increment to antibiotics requires solutions expanding from the discovery of new antibiotics, improving the clinical practice to innovative approaches [52,53,55,56]. Among the proven efficient approaches, the attenuation of bacterial virulence is considered a promising one [57,58]. Several studies have been conducted to mitigate bacterial virulence by a wide diversity of chemical and natural compounds [30,31,32,34,59]. Particularly, the anti-virulence activities of several approved safe drugs were investigated prior to their repurposing to serve as adjuvants to antibiotics [31,32,34]. In previous studies, adrenoreceptor antagonists were screened for their anti-QS and anti-virulence activities [31,32]. Interestingly, β-adrenoreceptor antagonists atenolol and metoprolol diminished the virulence of *Pseudomonas aeruginosa, Proteus mirabilis*, *Serratia marcescens, Salmonella enterica*, and *E. coli* [32,60,61]. In addition, α-adrenoreceptor blocker antagonist terazosin showed significant in vitro and in vivo reduction in *S. enterica* and *P. aeruginosa* pathogenesis [19,31]. Furthermore, the anti-QS activities of prazosin were screened against *P. aeruginosa*, *P. mirabilis*, *S. marcescens*; it diminished the biofilm formation and reduced the bacterial pathogenesis in vitro and in vivo [62]. As the virulence behavior of *S. enterica* is quite different from *P. aeruginosa*, *P. mirabilis*, and *S. marcescens*, the current study aimed to evaluate the anti-virulence activities of prazosin against *S. enterica* and *E. coli* K12, investigating the possible mechanisms and discovering more bacterial targets. The basis of this approach is diminishing bacterial virulence without affecting the growth that helps the immune system to completely kill the invaders without allowing them to develop resistance [10,35]. In this context and to avoid any effect of prazosin alone on bacterial growth, the anti-virulence activities of prazosin were evaluated at sub−MIC concentrations. Furthermore, the prazosin effect at sub−MIC on bacterial growth was evaluated, and there was no significant effect of prazosin at sub−MIC on the growth of tested strains. That ensures all the prazosin effects will be due to its interplay with bacterial virulence not due to inhibition of the growth.

*S. enterica* is one of the most clinically important gut pathogens that cause a wide array of infections from local gastroenteritis to systemic enteric fever [2,15]. However, chloramphenicol was known as the drug of choice for *S. enterica* infection, the increased resistance to it besides adverse effects limits its use [4,63]. Furthermore, the resistance increment to several other antibiotic classes was observed around the world constituting a problem for public health especially in developing countries [3]. *E. coli* K-12 is innately defective as a pathogen, but it represents the genetically best understood Enterobacteriaceae [64].

*Salmonella* employs QS systems to regulate its virulence such as motility, biofilm formation, and the production of virulence factors [5,6,9]. However, *Salmonella* does not produce its own AHLs autoinducers; it senses the AHLs produced by other species on Lux-analogs SdiA [6]. It is worthwhile to mention that a variety of AHLs with variable acyl chain lengths and C-3 substitutes are recognized by SdiA [5,6]. Askoura et al. showed the very essential role of SdiA in *S. typhimurium* adhesion and biofilm formation where the *sdiA* mutant strain was not able to adhere or form biofilm. Furthermore, they proved that the invasiveness and intracellular replication of *sdiA* mutants were significantly diminished in comparison to wild type [5]. In the current study, prazosin showed considered ability to alter SdiA, significantly downregulating the expression of the *sdiA* gene. In agreement with these findings, prazosin significantly diminished the *E. coli* and *S*. Typhimurium adhesion and biofilm formation.

Virulence genes are arranged in specific loci on the *S. enterica* chromosome called *Salmonella* Pathogenicity Islands (SPIs). However, several SPIs are recognized; SPI-1 and SPI-2 encode the expression of two types of T3SS [16,17]. The *S. enterica* T3SS is characterized by its injectosome-like structure, which injects the effectors to modulate its invasion and intracellular replication [13,14,17]. T3SS-type I translocates its effectors to modify the G-protein and cellular skeleton of the host cell facilitating the invasion in the early stages of infection [2,15,17]. After the entry of *Salmonella* into the host cells, they are engulfed in vacuole and phagosomes inside the immune cells. In this stage, the second type of T3SS translocates its effectors to permit *Salmonella* survival and intracellular replication inside the phagosome of immune cells [2,50]. While the present findings revealed the anti-QS activities of prazosin, it is worthwhile to draw the attention to roles of QS in the activation of the T3SS expression as reviewed in [65]. Furthermore, prazosin at sub−MIC significantly downregulated the expression of SPI-2 T3SS genes, which is responsible for *Salmonella* intracellular survival and replication. The intracellular replication of *sdiA* mutants was shown deficient in comparison to wild type [5,9]. In agreement with the downregulation of T3SS type II and *sdiA* downregulation, prazosin decreased the intracellular replication of *Salmonella* inside macrophages.

The interkingdom cross-talk is observed between bacterial and host cells, particularly gut pathogens such as Salmonella and *E. coli* K-12 [18,22]. *Salmonella* senses adrenergic hormones using sensor kinases such as QseC and QseE, resulting in enhancing the pathogenesis [20,21]. The norepinephrine effect on augmentation of the virulence of *Salmonella* in vitro and in vivo was documented [23]. Meanwhile, QS autoinducers (AIs) cross-talk with the adrenergic hormones, initiating the same signaling pathway in the host cells [20,21,22]. The AIs stimulate the production of norepinephrine in host cells that results in augmenting virulence [20,22]. Interestingly, prazosin showed a considered affinity to QseC competing with norepinephrine and downregulated the expression of *qseC* and *qseE* genes at its sub−MIC. In compliance with in vitro results, prazosin at sub−MIC significantly decreased the *S*. Typhimurium killing capacity against mice conferring 30% protection. The current findings support the efficient anti-virulence activities of prazosin against *S*. *typhimurium* and the potential to be used as adjuvant to antibiotics. The correlation between the stress and establishing of microbial infection in general was widely documented [66,67]. The release of adrenergic hormones was accompanied with induction of inflammation and vice versa is correct, as the microbial infection induces stress and inflammation, which results in an increase in adrenergic hormones release [66,68]. In addition to the prazosin anti-QS and anti-virulence effects, as an adrenergic receptor blocker, it will antagonize the norepinephrine, decreasing its induction to inflammation and bacterial virulence. Furthermore, the controlling of inflammation will greatly decrease the bacterial pathogenesis, and hence, the correlating between the use of prazosin and production of cytokine should be fulfilled before the clinical use of prazosin as anti-virulence. Despite the potent anti-virulence activities of prazosin, the new use as anti-virulence mandates further pharmacological investigation to evaluate the suitable route of administration and doses to reduce the adverse effects.

## 4. Materials and Methods

### 4.1. Chemicals and Microbiological Media

All the used chemicals were of pharmaceutical grade. The microbiological media were purchased from Oxoid (Hampshire, UK). N-hexanoyl-DL-homoserine lactone (AHL) (CAS Number: 106983-28-2), prazosin (CAS number: 19237-84-4), DL-norepinephrine hydrochloride (CAS Number: 55-27-6), thiamine (CAS number: 67-03-8) and Dulbecco’s Modified Eagle’s Medium (DMEM) medium were purchased from Sigma-Aldrich (St. Louis, MO, USA).

### 4.2. Bacterial Strains and Growth Conditions

*Salmonella enterica* serovar Typhimurium (NCTC 12023) and *Escherichia coli* K-12 MG1655 were used in this study. For *S*. Typhimurium, fresh overnight cultures were cultivated in Tryptic Soy Broth (TSB) or Luria–Bertani (LB) broth provided with 0.001 μM AHL [5]. For *E. coli* K-12, fresh overnight cultures were cultivated in AB minimal media [69] supplemented with 0.5% glucose and provided with 2.5 mg/mL thiamine [70]. The bacterial cell cultures were adjusted to cell density 1 × 10^6^ CFU/mL (OD600 = 0.4) prior to each experiment.

### 4.3. Determination of Minimum Inhibitory Concentrations (MICs) and Prazosin Effect on Bacterial Growth

The MICs of prazosin against *S*. *typhimurium* and *E. coli* K-12 were detected by the broth microdilution method according to the Clinical Laboratory and Standards Institute Guidelines (CLSI, 2020) [34].

The effect of prazosin at sub−MIC (1/4 MIC) on bacterial growth was examined as described earlier [35]. Briefly, the bacterial viable counts and optical densities of cultures provided or not with prazosin at 1/4 MIC was performed.

### 4.4. Quantitative RT-PCR

The RNA of prazosin at sub−MIC treated or untreated *S*. Typhimurium was isolated as formerly described [19,71]. The obtained RNA was used to synthesize cDNA, RT-PCR was performed to assess the genes’ expressions, and the relative expression was calculated by the comparative threshold cycle (^∆∆^Ct) method [72]. The used primers are listed in Table 1.

### 4.5. Evaluation of Biofilm Formation

The tested bacterial strains *S. typhimurium* and *E. coli* K-12 were cultivated in the growth conditions mentioned above prior to evaluation of the biofilm formation in the presence or absence of prazosin at sub−MIC. As described previously, the crystal violet method was used to quantify the biofilm formation [5]. For visualization of the prazosin inhibitory effect on biofilm formation, the bacterial biofilms were allowed to be formed on cover slips in the presence or absence of prazosin at sub−MIC, as described before [35].

### 4.6. Evaluation of the S. typhimurium Intracellular Replication

A gentamicin protection assay was used to evaluate the *S. Typhimurium* intracellular replication within macrophages in the presence or absence of prazosin at sub−MIC, as previously described [5,16]. Briefly, 24-well polystyrene plates were seeded with macrophages RAW264.7 at cell densities of 2 × 10^5^ cells/well. *S. Typhimurium* were grown overnight in the LB broth provided with 0.001 μM AHL in the presence or absence of prazosin at sub−MIC, and then, fresh overnight cultures were incubated with shaking for 3 h at 37 °C. A master-mix (1 × 10^5^ bacteria/well) was prepared with multiplicity of infection (MOI 1) in DMEM and distributed in wells. The non-internalized *S. Typhimurium* cells were washed out with pre-warmed phosphate buffer saline (PBS) after a half hour. Furthermore, gentamicin (100 μg/mL) was used to kill extracellular adhered *S. Typhimurium* cells for 1 h. The infected macrophages were washed with PBS and lysed with TritonX-100 (0.1%) for 20 min at room temperature at 2 and 16 h post infection. The initial inoculum and the lysates were viably counted onto MH plates. The phagocytosed cell numbers/relative untaken cells (2 h against inoculum) and x-fold intracellular replication (16 h against 2 h) were calculated.

To visualize the Salmonella-infected macrophages, bacterial cells were immune-stained as earlier described [4,5]. The macrophage infection was performed as described above; then, the infected macrophages were fixed with 2% paraformaldehyde for 30 min. After washing with PBS, 2% of bovine serum albumin (BSA) was used as a blocking agent for 1 h. After washing with PBS, rabbit anti-Salmonella O antigen (Difco, BD; San Joes, CA, USA) were added to stain Salmonella cells for 4 h; then, they were washed with PBS. Anti-rabbit tagged with green fluorescent protein (GFP) secondary antibody (green fluorescent protein) (Abcam; Eugene, OR, USA) was added for 1 h. Finally, the macrophages were counter-stained with blue fluorescent diamidino-2-phenylindole dye (DAPI) (Thermo Fisher Scientific; Bothell, WA, USA) for 1 h, then washed with PBS. A LSM780 confocal laser scanning microscope (Carl Zeiss, Jena, Germany) was used to capture images.

### 4.7. Evaluation In Vivo Anti-Virulence Activity

The mice survival model was employed to assess the in vivo anti-virulence activity of prazosin against *S. Typhimurium*, as formerly described [19,31]. Briefly, adjusted overnight S. Typhimurium cultures (1 × 10^6^ CFU/mL) were treated or not with prazosin at sub−MIC in PBS. Twenty female Mus three-week-old musculus BALB/c mice were distributed in two groups (n = 10) to be intraperitoneally (ip) injected with 100 μL *S. Typhimurium* treated or not with prazosin. Another mice group was injected with *S. Typhimurium* treated with norepinephrine (50 μg/mL). Two mice groups were injected with sterile PBS or kept uninfected. Death and survival were recorded for 5 days using the Kaplan–Meier method. To comply with the ARRIVE guidelines and in accordance with the U.K. Animals (Scientific Procedures) Act 1986 and associated guidelines; at the end of the experiment, mice suffered from pathological conditions, and/or loss of weight and appetite were anesthetized by thiopental and euthanized by cervical dislocation.

### 4.8. Statistical Analysis

Student’s *t*-test was used to evaluate the statistical significance (unless mentioned), where *p* value < 0.05 is considered significant (GraphPad Prism Software, v.8, San Diego, CA, USA). The assessments were performed in triplicate, and the data are presented as the means ± standard error.

## 5. Conclusions

S. enterica is one of the most clinically important gut pathogens. S. enterica employs QS systems to regulate its virulence and to activate its T3SS. Additionally, S. enterica eavesdrop on the host cells, sensing noradrenergic hormones to facilitate its infection and enhance pathogenesis. The present findings preliminary evaluated the in vitro and in vivo anti-virulence activities of prazosin against S. Typhimurium. Prazosin downregulated the QS receptor SdiA and norepinephrine sensor QseC encoding genes. Prazosin diminished the bacterial adhesion and biofilm formation and protected the mice against S. typhimurium. Furthermore, prazosin downregulated the SPI2-T3SS encoding genes and decreased the intracellular replication of S. typhimurium in macrophages. These findings support the potent anti-virulence activities of prazosin to be used in treatments of resistant S. typhimurium infections; however, it requires further pharmacological assessments before its clinical application.

## Figures and Tables

**Table 1:** The primers used in this study.

**Target Gene**	**Primer Sequence: 5′- 3′**	**Gene Significance**	**Reference**
*gyrB*	F: GTGATCAGCGTCGCCACTR: GCGCGGTGATCAGCGTC	Housekeeping	[4]
*sdiA*	F: AAT ATC GCT TCG TAC CACR: GTA GGT AAA CGA GGA GCA G	Adhesion	[73]
*qseC*	F: GGTACCAAATTGACGCAACGTCTCAGR: GAATTCGCCCAACTTACTACGGCCTC	Sensor to adrenergic hormones	[20]
*qseE*	F: GGTACCAGCGACACGTTGAAGCGCR: GAATTCGCGTGTTTGTCAGATGCAGG	Sensor to adrenergic hormones	[20]
*ssrB*	F: CGCAGGTGCTAATGGCTATGR: TTTGCAATGCCGCTAACAGA	SPI2-expression regulation	[4]
*ssaE*	F: CCGCAGCAATATCAGCAAAAR: AAGTGCGCTGTTATGGTAACGA	SPI2-intracellular replication	[4]
*ssaJ*	F: TGTCGAGCAGTCGCAGTTTATTAR: TGCCTATGCGGATAACCGTTA	SPI2-intracellular replication	[4]
*sseF*	F: TCAGGAATCGCTATTTCTATGR: GTCAGGCTAACGGAGGTAA	SPI2-intracellular replication	[4]
*sseJ*	F: AATAAATCACATCCCAAGCR: ACTCAGTCCAGGTAAATCC	SPI2-intracellular replication	[4]
*sseI*	F: GATACCCCCCCTGAAATGAGTTR: GTGACAAATCGTCCAGATGCA	SPI2-intracellular replication	[4]
*sifA*	F: TACCACCACCGCATACCCAR: ACGAGGAACGCCTGAAACG	*Salmonella*-inducing filaments (SPI2)	[4]
*SifB*	F: TGATACTCAGCCTGCCCACR: GCTCAGGGAACAAGCAAC	*Salmonella*-inducing filaments (SPI2)	[4]
*sscA*	F: GGCTCGCTGCGTATGTTGTTR: GCCGGCGAATTCTTTTACCT	SPI2 chaperon intracellular replication	[4]
*qseC*	F: GGTACCAAATTGACGCAACGTCTCAGR: GAATTCGCCCAACTTACTACGGCCTC	Sensor to adrenergic hormones	[20]
*qseE*	F: GGTACCAGCGACACGTTGAAGCGCR: GAATTCGCGTGTTTGTCAGATGCAGG	Sensor to adrenergic hormones	[20]

## Data Availability

All data included in the main text.
